# An Atypical Presentation of Pityriasis Rosea Localized to the Extremities

**DOI:** 10.7759/cureus.9765

**Published:** 2020-08-15

**Authors:** Robert P Daze, David Dorton

**Affiliations:** 1 Dermatology, Largo Medical Center, Largo, USA

**Keywords:** pityriasis rosea, papulosquamous, hhv-6, hhv-7

## Abstract

Pityriasis rosea (PR) is a benign, self-limiting, papulosquamous disorder characterized by the onset of a herald patch followed by an abrupt eruption of multiple salmon-colored papules and plaques on the trunk and proximal extremities. The individual lesions are often outlined by a collarette of scale and are distributed along the lines of cleavage. While many authors acknowledge an etiologic association with human herpesvirus 6 and human herpesvirus 7, thereby suggesting a viral exanthem, the exact cause remains unclear. While typically demonstrating a truncal predilection, this presentation may be absent in some patients who instead exhibit atypical features and distributions. Various clinical variants include papular, vesicular, purpuric, and eczematoid. The condition rarely manifests without truncal involvement and localized only to the distal extremities. We present a unique case report of a 65-year-old-male with biopsy-proven PR that was localized to his distal extremities with a clinical absence of truncal involvement.

## Introduction

Pityriasis rosea (PR) is a self-limited cutaneous eruption that typically presents with the onset of a pink to salmon-colored herald patch on the trunk or proximal extremities followed by an acute dissemination of multiple oval scaly patches and plaques along skin cleavage lines [1]. While the etiology remains unclear, the current literature suggests a systemic reactivation of human herpesvirus 6 and 7 [1-2]. Certain drugs are also associated with pityriasiform lesions. Atypical variants have been described and include the following: unilateral, inverse, vesicular, papular, urticarial-like, erythema multiforme-like, and purpuric [3]. Since atypical presentations can be clinically challenging, the physician should keep in mind several other papulosquamous eruptions for diagnostic consideration as it will ultimately affect subsequent management. PR does not require treatment. However, there are therapies available to help with symptoms and to possibly hasten clearance.

## Case presentation

**Figure 1:**
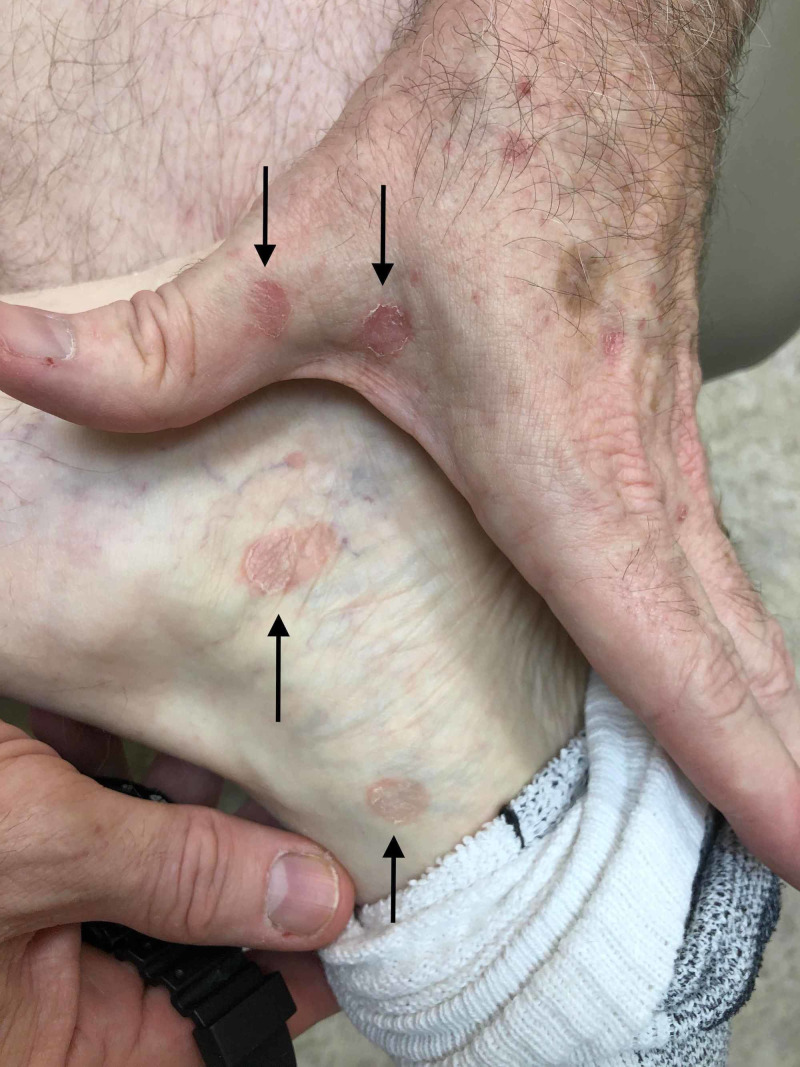
Multiple, discrete pink to erythematous scaly macules distributed on the left dorsal hand and medial aspect of the right foot (black arrows)

**Figure 2:**
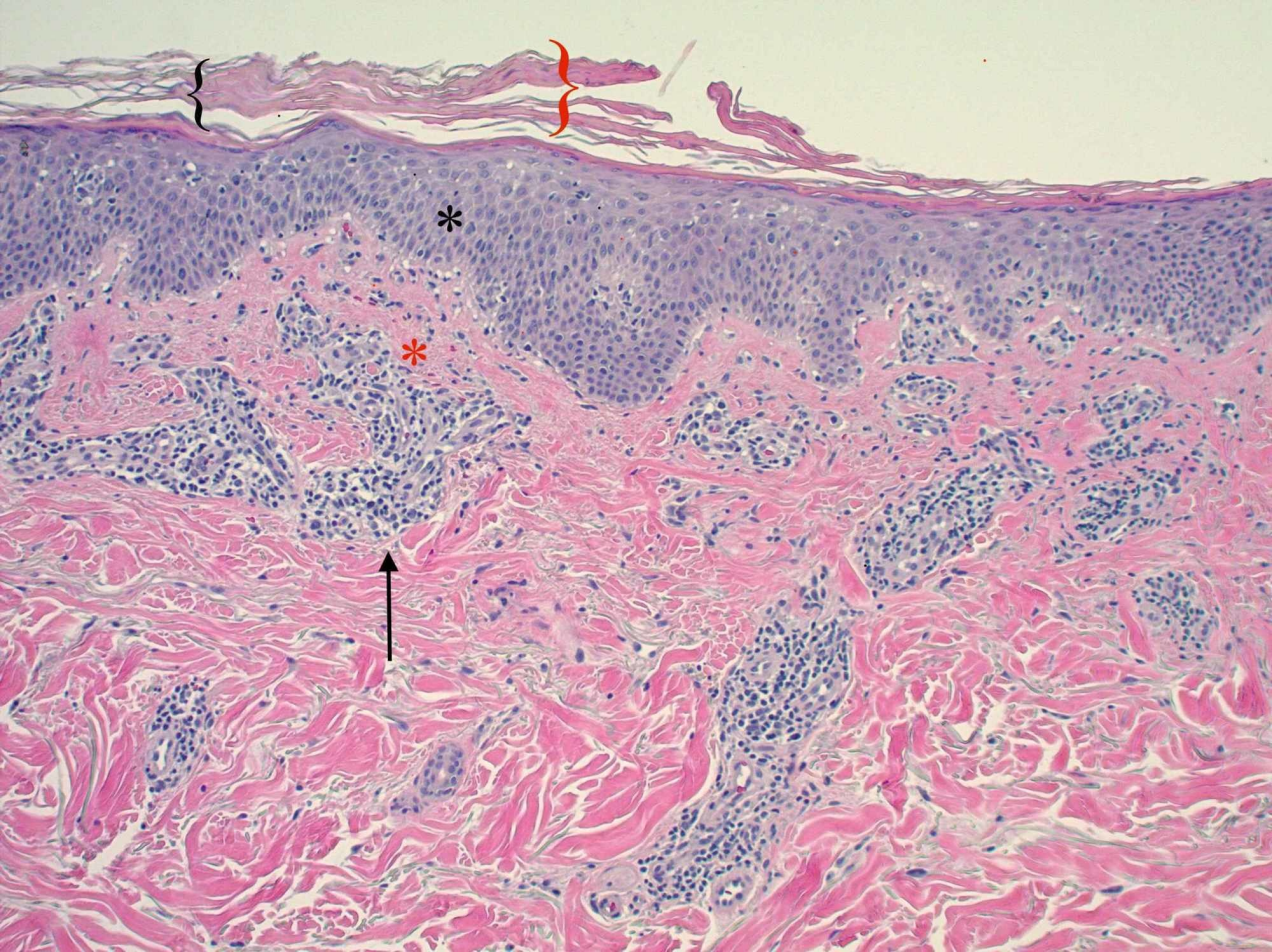
Lesional punch biopsy specimen (hematoxylin-eosin, original magnification x 100) The image shows focal hyperkeratosis (black bracket), angulated parakeratosis (red bracket), mild spongiosis (black asterisk), extravasated red blood cells (red asterisk), and a lymphohistiocytic infiltrate surrounding vessels (black arrow)

**Figure 3:**
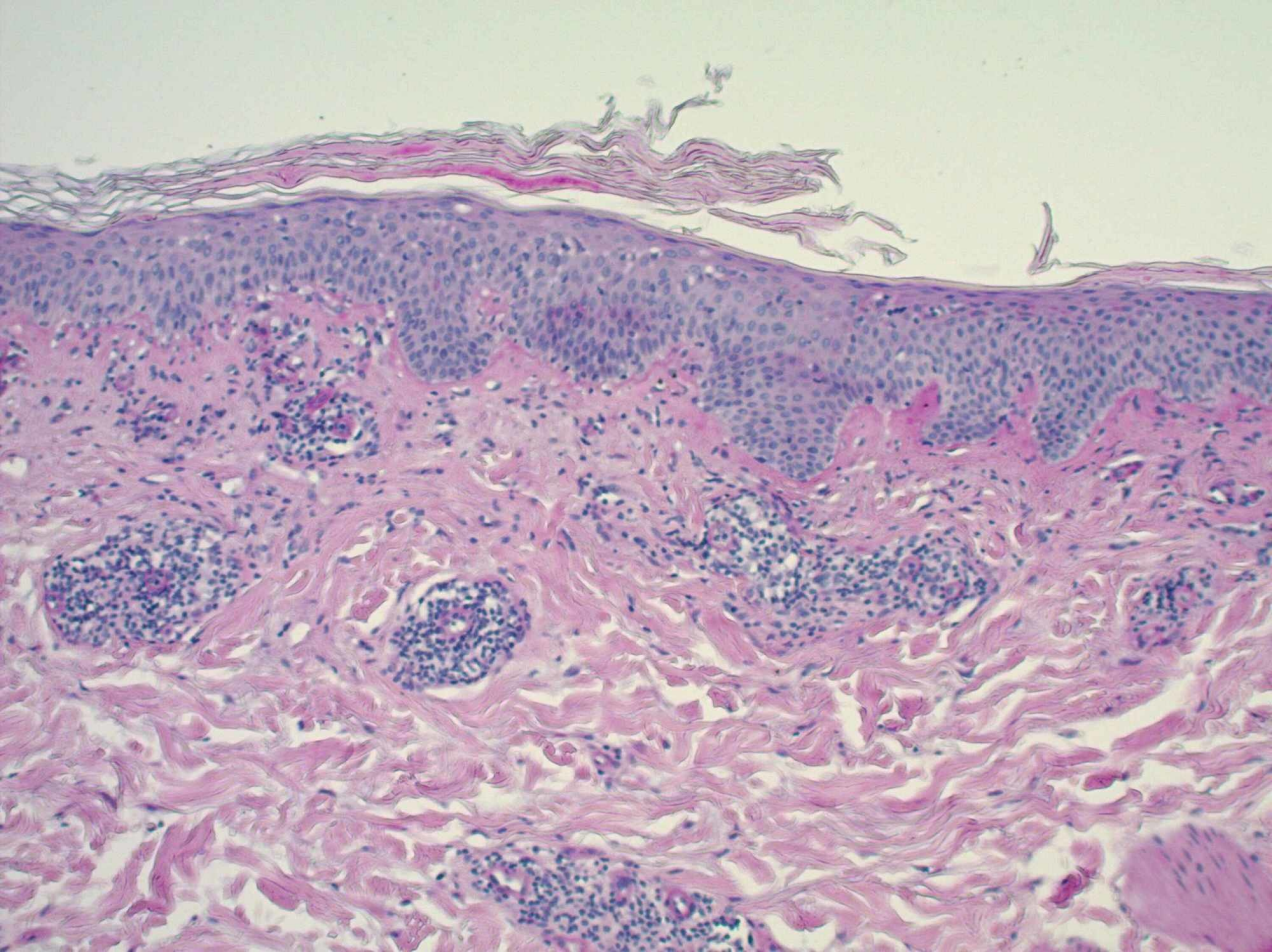
Lesional punch biopsy specimen (Periodic Acid-Schiff, original magnification x 100) The image shows no evidence of fungal elements

**Figure 4:**
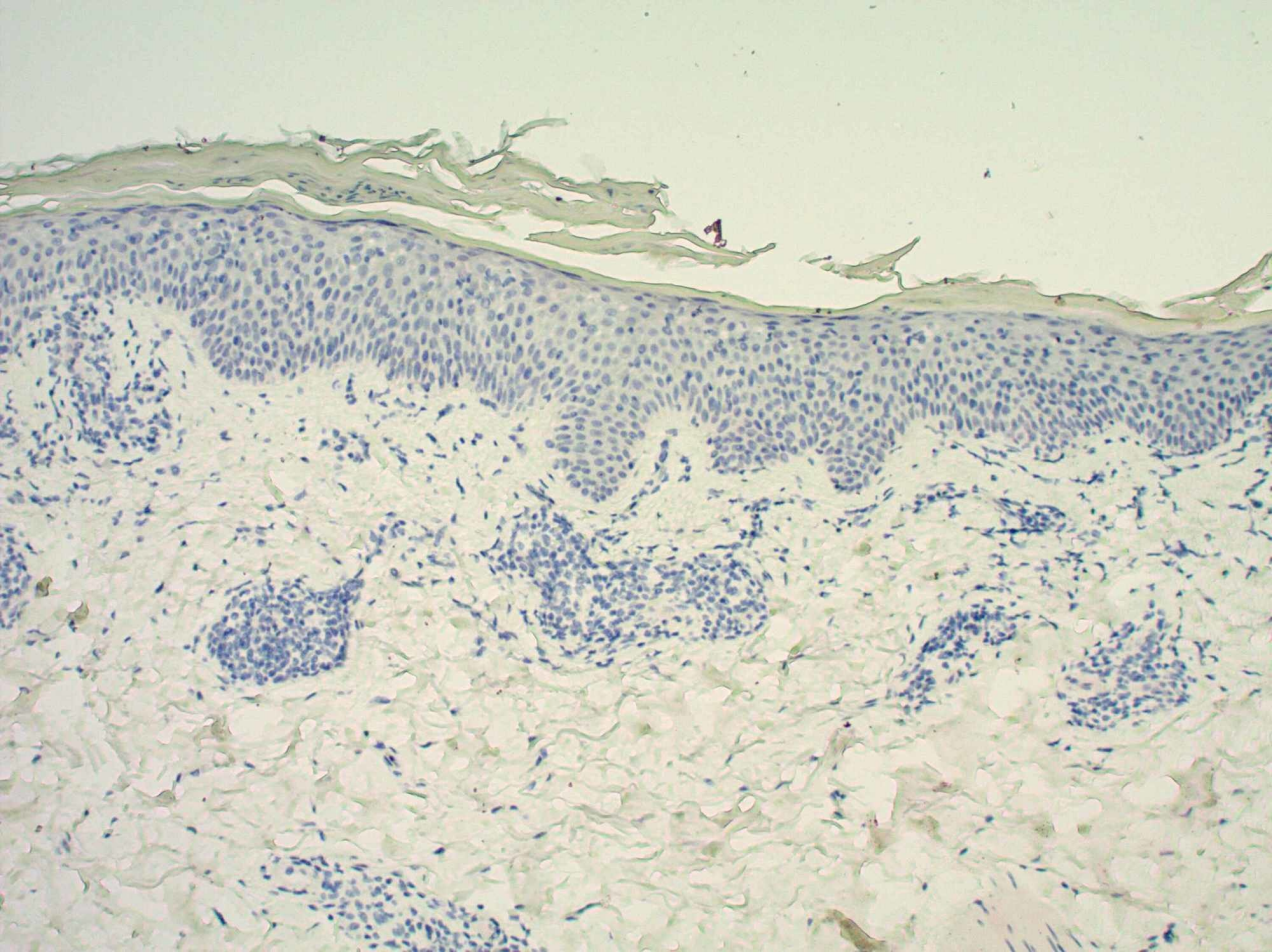
Lesional punch biopsy specimen (anti-Treponema pallidum immunohistochemical stain, original magnification x 100) The procedure was negative for treponemal organisms

A 65-year-old Caucasian male with a past medical history significant for bladder cancer, who was undergoing chemotherapy with the last treatment session a few months prior, presented for the evaluation of multiple, scattered, scaly lesions present on his distal bilateral upper and lower extremities that had started one month prior to the presentation in the winter (Figure 1). He had noticed an eruption that started on the interdigital fold between the thumb and index finger of his left hand. Around the same time, he had identified similar lesions present on the medial aspect of his right dorsal foot with subsequent development of scaly papules involving the ventral surface of his forearms. The patient denied any symptomatology including pruritus, pain, or dysesthesias. He also denied any prodromal symptoms prior to the onset of the present condition. A review of systems was negative for any systemic involvement. No alleviating or exacerbating factors were noted. The patient denied any recent medication changes. A 4-mm punch biopsy was performed with supplemental stains including Periodic Acid-Schiff (PAS) for fungi and an immunohistochemical stain for *Treponema pallidum*. Histopathological assessment of the lesion demonstrated spongiotic dermatitis with extravasation of red blood cells and focal parakeratosis (Figure 2). PAS stain was negative for pathogenic fungi (Figure 3), and the *Treponema pallidum* immunohistochemical stain was negative for treponemal organisms (Figure 4). Additionally, a rapid plasma reagin was non-reactive, confirming the absence of syphilis. The above clinical and histopathological correlation confirmed the diagnosis of PR. The patient was re-evaluated four weeks after the clinical presentation. Due to marked clinical improvement, no further testing, diagnostic workup, or treatment was required.

## Discussion

PR is a benign, self-limiting papulosquamous disorder with the majority of cases affecting adolescents and young adults between the ages of 10 and 35 years with a seasonal predilection for the spring and fall [1]. The etiology remains a point of debate in the literature. However, a viral etiology secondary to the human herpesvirus 7 and human herpesvirus 6 has been proposed [1,2]. The eruption lasts anywhere between six to eight weeks, but recurrent and persistent variants have also been documented [3]. In its classical form, a solitary ovoid patch presents on the trunk, which represents the “herald patch” as it marks the onset of the disease [1]. Typically, the advancing margin of this patch will present with a collarette of scale. A secondary eruption develops with numerous smaller plaques on the trunk and proximal extremities following the Langer cleavage lines. On the posterior trunk, this distribution is referred to as the “Christmas tree” pattern [1]. The main histological patterns include epidermal hyperplasia, spongiosis, focal parakeratosis in mounds, a superficial perivascular inflammatory infiltrate, and a variable amount of extravasated red blood cells [1].

Case reports documenting acral involvement both in the pediatric and adult populations are scarce, indicating that such a presentation is exceedingly rare [4-7]. Zawar has delineated an infantile presentation of PR limited to the acral surfaces without truncal involvement [4]. The other reported cases of pediatric and adult acral PR have involved the trunk [5-7]. Our case further emphasizes the clinical rarity of acral PR isolated to the distal extremities.

While the classic presentation is easily recognized, unusual presentations of PR exist, making it a diagnostic challenge that requires histopathological assessment to rule out other mimickers such as dermatophytosis, pityriasis lichenoides chronica (PLC), syphilis, and guttate psoriasis. A dermatophyte infection should be considered in cases presenting with epidermal scaling. However, the PAS stain was negative in our case, and therefore, a dermatophyte infection was ruled out. PLC is typified by an interface change and vacuolar degeneration of the basal layer of the epidermis, which are not seen in PR [8]. Clinically distinguishing between syphilis and PR can be difficult. Nevertheless, several histological features are strongly associated with syphilis and favor this diagnosis over PR: neutrophils in the stratum corneum, plasma cells, vacuolar interface change with lymphocytes, and lymphocytes with ample cytoplasm [8]. According to the US Preventative Services Task Force, a rapid plasma reagin test can be ordered to rule out secondary syphilis with 100% sensitivity and 85-99% specificity [9].

Guttate psoriasis demonstrates significant clinical overlap with PR. However, a distinguishing characteristic favoring psoriasis is the presence of neutrophils within the parakeratotic mounds, otherwise known as Munro microabscesses [10]. Other clinical mimickers to consider include small plaque parapsoriasis and erythema annulare centrifugum. Small plaque parapsoriasis presents with multiple, small, scaly patches, but PR is distinguished by the presence of a herald patch and spontaneous resolution [1]. Erythema annulare centrifugum shares the trailing scale of PR, but histopathologically, erythema annulare centrifugum may present with a dense lymphohistiocytic infiltrate surrounding superficial vessels, known as the “coat-sleeve appearance” [11].

Similarly, PR-like eruptions have been described in the literature and multiple drugs have been implicated: captopril, gold, isotretinoin, non-steroidal anti-inflammatory agents, omeprazole, terbinafine, and tyrosine kinase inhibitors [12]. Clinically, a PR-like eruption will lack a herald patch, and the histopathologic findings would be consistent with an interface dermatitis with eosinophils [13]. 

As this condition exhibits a benign nature, patients should be reassured that most cases require no treatment and that there are no permanent sequelae. Current therapies are directed at symptomatic relief, and they include topical corticosteroids, emollients, and oral antihistamines [1]. The use of oral erythromycin in a double-blind, placebo-controlled trial has demonstrated resolution in 73% of patients as compared to the placebo group with no resolution [14]. Case reports have noted clinical improvement with the use of acyclovir given the possible viral etiology [3]. 

PR is a relatively common disease with the majority of cases presenting with classic morphology and distribution. It is pertinent for the dermatologist to recognize the typical and atypical presentations of PR in order to avoid diagnostic pitfalls. Multiple variants have been cited in the literature and include acral, inverse, purpuric, papular, follicular, vesicular, and oral [3]. The purpose of this article was to document a very rare manifestation of PR, which was localized to the patient’s distal extremities without any truncal involvement.

## Conclusions

In conclusion, acral presentations of PR in the absence of truncal involvement are exceedingly rare. However, despite its rarity, clinicians should approach this atypical eruption with appropriate diagnostic modalities in consideration of other papulosquamous dermatoses. PR can present in many different forms and localizations. Our case report highlights the importance of a clinical and histopathological correlation to aid in the diagnosis of this unique presentation.
